# Impact of Age and HIV Status on Immune Activation, Senescence and Apoptosis

**DOI:** 10.3389/fimmu.2020.583569

**Published:** 2020-09-30

**Authors:** Malene Hove-Skovsgaard, Yanan Zhao, Jeanette Linnea Tingstedt, Hans Jakob Hartling, Rebekka Faber Thudium, Thomas Benfield, Shoaib Afzal, Børge Nordestgaard, Henrik Ullum, Jan Gerstoft, Amanda Mocroft, Susanne Dam Nielsen

**Affiliations:** ^1^Department of Infectious Diseases, Rigshospitalet, University of Copenhagen, Copenhagen, Denmark; ^2^Department of Clinical Immunology, Rigshospitalet, University of Copenhagen, Copenhagen, Denmark; ^3^Department of Infectious Diseases, Hvidovre Hospital, Copenhagen University Hospital, Amager and Hvidovre, Hvidovre, Denmark; ^4^Faculty of Health and Medical Sciences, Institute of Clinical Medicine, University of Copenhagen, Copenhagen, Denmark; ^5^The Copenhagen General Population Study, Department of Clinical Biochemistry, Herlev and Gentofte Hospital, Copenhagen University Hospital, Herlev, Denmark; ^6^Centre for Clinical Research, Epidemiology, Modelling and Evaluation (CREME), Institute for Global Health, UCL, London, United Kingdom

**Keywords:** HIV infection, flow cytometry, T-cells, immune activation, immune senescence, aging

## Abstract

**Introduction:**

Residual immune dysfunctions, resembling those that occur during normal aging, may persist even in well-treated people with HIV (PWH), and accelerated aging has been proposed. We aimed to determine if HIV infection is an independent risk factor for T-cell immune dysfunctions including increased immune activation, senescence and apoptosis. Moreover, in PWH we aimed to identify the associations between age and immune activation, senescence and apoptosis.

**Materials and Methods:**

We included 780 PWH with suppressed viral replication (<50 copies/mL) and absence of hepatitis B and hepatitis C co-infection and 65 uninfected controls from the Copenhagen Co-morbidity in HIV Infection (COCOMO) Study. Flow cytometry was used to determine T-cell activation (CD38+HLA-DR+), senescence (CD28-CD57+), and apoptosis (CD28-CD95+). T-cell subsets are reported as proportions of CD4+ and CD8+ T-cells. We defined an elevated proportion of a given T-cell subset as above the 75th percentile. Regression models were used to determine the association between HIV status and T-cell subset and in PWH to determine the association between age or HIV-specific risk factors and T-cell subsets. Furthermore, an interaction between HIV status and age on T-cell subsets was investigated with an interaction term in models including both PWH and controls. Models were adjusted for age, sex, BMI, and smoking status.

**Results:**

In adjusted models a positive HIV status was associated with elevated proportions of CD8+ activated (*p* = 0.009), CD4+ senescent (*p* = 0.004), CD4+ apoptotic (*p* = 0.002), and CD8+ apoptotic (*p* = 0.003) T-cells. In PWH a 10-year increase in age was associated with higher proportions of CD4+ and CD8+ senescent (*p* = 0.001 and *p* < 0.001) and CD4+ and CD8+ apoptotic T-cells (*p* < 0.001 and *p* < 0.001). However, no interaction between HIV status and age was found. Furthermore, in PWH a CD4+/CD8+ ratio < 1 was associated with elevated proportions of T-cell activation, senescence, and apoptosis.

**Discussion:**

We found evidence of residual T-cell immune dysfunction in well-treated PWH without HBV or HCV co-infection, and age was associated with T-cell senescence and apoptosis. Our data supports that HIV infection has similar effects as aging on T-cell subsets. However, since no interaction between HIV status and age was found on these parameters, we found no evidence to support accelerated immunological aging in PWH.

## Introduction

In untreated persons with HIV (PWH), viral replication and antigen exposure contribute to sustained immune activation which, in turn, is found to be an independent predictor of CD4+ T-cell depletion and progression to AIDS (1, 2). Combination antiretroviral treatment (cART) suppress viral replication and restores CD4+ T-cell counts in the majority of PWH and has reduced AIDS-related morbidity and mortality (3, 4). Nevertheless, the estimated life expectancy for PWH is lower than in the background population, and a growing body of evidence suggests that PWH have excess risk of age-related comorbidities (3, 5–7). The excess risk seems partly to be attributable to a greater burden of traditional risk factors such as smoking and obesity, but residual immune dysfunction is thought to contribute to the pathogenesis as well.

Importantly, despite CD4+ T-cell recovery, residual immune dysfunctions including immune activation, senescence, and apoptosis have been reported to be features of treated HIV infection (8–10) possibly due to low grade viral replication, microbial translocation, co-infections with hepatitis C virus (HCV), hepatitis B virus (HBV), or cytomegalovirus (CMV) (11–13). However, T-cell activation, senescence, and apoptosis, in PWH may also be affected by lifestyle factors such as smoking and obesity (14, 15). Therefore, it is debated if residual immune dysfunction in well-treated PWH is caused by HIV-specific risk factors, co-infections or by traditional risk factors. The residual immune dysfunctions in PWH resemble what is found in normal aging where markers of T-cell senescence are associated with shorter telomere length, replicative senescence, and inflammation (16, 17). Concomitant immune dysfunction and increased risk of age-related comorbidity have led to the hypothesis that HIV infection accelerates the aging process including immunological aging, a phenomenon known as “immunosenescence” (18).

Previous studies of residual immune dysfunctions in PWH have been small, lacked uninfected controls or included PWH with ongoing viral replication, or with chronic HBV or HBC infection. Thus, it is unknown if residual T-cell immune activation, senescence and apoptosis are features of well-treated HIV infection. Furthermore, the impact of aging on residual immune dysfunctions in PWH is not well described.

In this study, we included a large cohort of well-treated PWH with undetectable viral replication and absence of HBV and HCV infection and a group of age matched uninfected controls. We aimed to determine if HIV infection is an independent risk factor for having T-cell immune activation, senescence and apoptosis. Moreover, in PWH we aimed to identify the impact of age on T-cell immune activation, senescence, and apoptosis. We hypothesized that PWH have higher T-cell immune activation and senescence than controls, and that immune activation and immune senescence would be more pronounced with increasing age.

## Materials and Methods

### Study Populations

Participants were included from the Copenhagen Co-morbidity in HIV Infection (COCOMO) Study. The COCOMO study is a longitudinal cohort study with the aim of assessing the burden of non-AIDS comorbidities in PWH that was initiated in March 2015 (19). Data presented in the current study are from the study baseline and the design is cross-sectional. The procedures for recruitment and data collection have been described in detail elsewhere (19). In brief, a comprehensive questionnaire on traditional risk factors including smoking and a physical exam including weight and height were performed at inclusion. Information regarding HIV-specific factors were obtained from medical records at inclusion. The uninfected controls also participated in The General Population Study (CGPS) (20). In total 780 PWH and 65 uninfected controls met the inclusion criteria that were age ≥18 years and peripheral blood mononuclear cells (PBMC) available for flow cytometry analyses. For PWH further inclusion criteria were a positive HIV test, treatment with cART, undetectable viral replication (<50 copies/mL), and no evidence of acute or chronic HBV or HCV infection.

Analyses comparing T-cell subsets in PWH and controls were done in a subset of PWH. Due to different age distribution in PWH and uninfected controls, a matching on age was done 5:1 (PWH *n* = 325 and uninfected controls *n* = 65).

The COCOMO study (NCT02382822) has been approved by the Committee on Health Research Ethics of the Capital Region of Denmark (H-8-2014-004) and the Danish Data Protection Agency. Written informed consent was obtained from all participants.

### Laboratory Analyses

#### Collection of PBMC

Blood was collected in heparin tubes [Becton Dickinson (BD), Franklin Lakes, NJ, United States]. Heparin blood was mixed 1:1 with Phosphate Buffered Saline (PBS, Sigma-Aldrich, Merck KGaA, Darmstadt, Germany), and PBMC were isolated by means of density gradient centrifugation using Leucosep^TM^ tubes (*in vitro* AS, Fredensborg, Denmark). Isolated PBMC were frozen in 1.8 mL Nunc Tubes (Thermo Fischer Scientific, Waltham, MA, United States) in 10% Dimethyl sulfoxide (DMSO) (WAK-Chemie, Steinbach, Germany), 40% Roswell Park Memorial Institute Medium (RPMI) (*in vitro* AS) and 40% Fetal Bovine Serum (FBS, Sigma-Aldrich) at a concentration of 10 × 10^6^ cells/mL. The last procedure was done at 4°C. PBMC were frozen using CoolCell^®^ cell freezing containers (BioCision, CA, United States) and moved to liquid nitrogen after 24–72 h.

#### Flow Cytometry Analyses

Peripheral blood mononuclear cells were thawed in 37°C water, resuspended in RPMI with 10% FBS and incubated overnight at 37°C and 5% CO_2_. Viability was found to be median (IQR) 75% (69–87). PBMC were washed and resuspended in RPMI with 10% FBS at a concentration of 1 × 10^6^/mL. Then 500 μL cells suspension, 50 μL BD Horizon^TM^ Brilliant Stain Buffer (BD), and antibodies were mixed in two tubes. Tube 1 to determine T-cell activation and tube 2 to determine T-cell senescence and apoptosis. The tubes were incubated for 20 min at room temperature protected from light. Afterward, PBMC were washed twice and resuspended in PBS. Monoclonal antibodies used to determine T-cell subsets were CD3+ Peridinin-chlorophyll proteins (PerCP, clone SK7), CD4+ Brilliant Violet 510 (BV510, clone SK3), CD8 Fluorescein isothiocyanate (FITC, clone HIT28a), CD28+ phycoerythrin-cyanine (PE-CY7, clone CD28.2), CD57+ Allophycocyanin (APC, clone NK-1), CD95+ Brilliant Violet 421 (BV421, clone DX2), CD38+ PE-Cy7 (clone HIT2), and HLA-DR+ APC (clone G46-6). All antibodies were purchased from BD. Fixable Viability Stain 780 (FVS780) (BD) was used to determine viability. Acquisition was done using a BD FACSCanto II.

#### Gating Strategy

**FIGURE 1:**
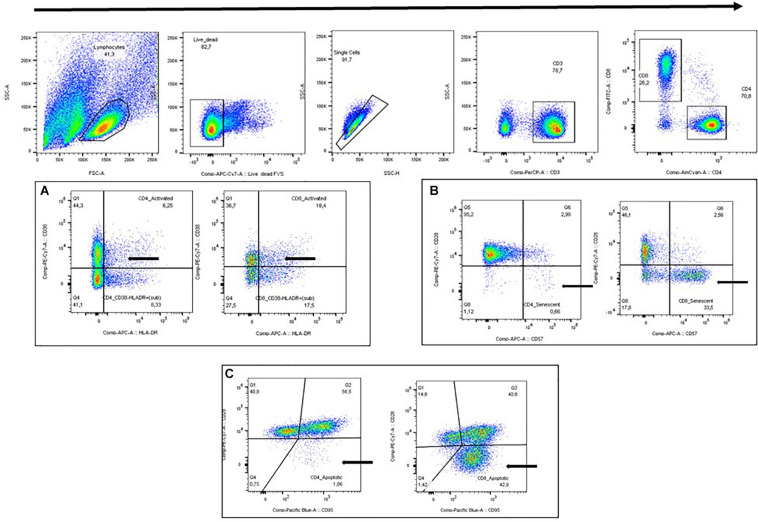
Gating strategy. **(A)** Activated T-cells (HLA-DR+ CD38+). **(B)** Senescent T-cells (CD28-CD57+). **(C)** Apoptotic T-cells (CD28-CD95+).

Forward and side scatter plot (FSC/SSC) was used to identify lymphocytes followed by viability and single cell plots. CD3+ was used in combination with CD4+ or CD8+ to identify activated T-cells (HLR-DR+ CD38+), senescent T-cells (CD28-CD57+), and apoptotic T-cells (CD28-CD95+) (Figure 1). FMO controls were performed, and the final gaiting strategy was based on both FMO controls and visual gating. All T-cell subsets were gated by the same operator using FlowJo v. 10.3 (BD). T-cell subsets are given as the proportion (%) of CD4+ T-cells or CD8+ T-cells, respectively.

### Statistical Analyses

Clinical characteristics are presented as median (IQR) or as proportions (%). Chi-square test was used for categorical variables and Wilcoxon’s rank test for continuous variables for comparison between PWH and uninfected controls. Proportions of T-cell subsets were presented as median and differences between PWH and uninfected controls were assed using Wilcoxon’s rank-sum test.

There is no established upper limit of normal for T-cells subsets. We defined elevated proportions of activated, senescent and apoptotic T-cells *a priori* using a cut-off value of >75th percentile defined from the entire study population (780 PWH and 65 uninfected controls). Logistic regression models were used to investigate the association between HIV status and having elevated proportions (i.e., >75th percentile) of a given T-cell subsets. The models were adjusted for potential confounders including age, sex, BMI group (underweight, normal, overweight, and obese) defined according to World Health Organization (WHO) criteria (21) and smoking status (former, current, or never smoker). Covariates were selected *a priori* based on current literature. Each T-cell subset was included in the model separately. We performed linear regression models to evaluate the association between age and T-cells subsets in PWH adjusting for sex, BMI group, and smoking status. Sensitivity analyses were done adjusting for nadir CD4+ and time on cART. Moreover, in a similar model including both PWH and uninfected controls, an interaction term between HIV status and age was added to the model. In the linear regression models T-cells subsets were log transformed using the natural logarithm prior to analyses to obtain normal distribution. Thus, results are shown in %.

Finally, in analyses restricted to PWH the association between HIV-specific factors including CD4+/CD8+ ratio, nadir CD4+, and CMV IgG and elevated proportions of the included T-cell subsets were investigated using logistic regression models adjusted for age, sex, BMI group and smoking status. The HIV-specific factors were included in the model one at a time. Both unadjusted and adjusted results are presented. *P*-values < 0.05 were considered significant. We did not adjust for multiple comparisons, but a statistical analysis plan predefining dependent and independent variables and models was made before study initiation. All statistical analyses were performed using R version 6.3.1.

## Results

### Demographic

A total of 780 PWH and 65 uninfected controls met the inclusion criteria and were included in the study. Characteristics of the participants are shown in Table 1. For analyses comparing proportions of T-cell subsets in PWH with uninfected controls a subgroup of 325 PWH that were age matched to uninfected controls were used. In the age matched subgroup of PWH, higher proportions were males (*p* = 0.005) and current smokers (*p* = 0.014) than in uninfected controls.

**TABLE 1:** Clinical characteristics.

	**PWH**	**PWH (matched on age)**	**Uninfected controls**
n	780	325	65
Age, median (range)	50.1 (21–85)	60.4 (34–85)	61.4 (34–80)
Sex, male (%)	660 (84.6)	283 (87.1)	47 (72.3)
BMI group (%)			
Underweight	24 (3.1)	9 (2.8)	0 (0.0)
Normal	413 (53.0)	174 (53.5)	27 (41.5)
Overweight	259 (33.2)	106 (32.6)	24 (36.9)
Obese	79 (10.1)	35 (10.8)	11 (16.9)
Unknown	5 (0.6)	1 (0.3)	3 (4.61)
Smoking status (%)			
Never smoker	258 (33.1)	86 (26.5)	19 (29.2)
Current smoker	222 (28.5)	86 (26.5)	4 (6.2)
Former smoker	274 (35.1)	144 (44.3)	24 (36.9)
Unknown	26 (3.3)	9 (2.7)	18 (27.7)
HIV-specific factors			
Current CD4+ count, median (IQR) (cells/μ L)	700 (540–890)	700 (530–898)	NA
Current CD8+ count, median (IQR) (cells/μ L)	840 (630–1170)	821 (503–1175)	NA
CD4+/CD8+ ratio, median (IQR)	0.8 (0.6–1.1)	0.8 (0.6–1.2)	NA
CD4+ nadir ≤200 (%) (cells/μ L)	317 (41.4)	159 (49.8)	NA
Time on cART (year), median (IQR)	13.6 (7.0–21.0)	18.3 (11.2–25.0)	NA
Earlier AIDS defining events (CDC) (%)			
0	644 (82.6)	258 (79.4)	NA
1	132 (16.9)	65 (20.0)	NA
2	1 (0.1)	1 (0.3)	NA
Unknown	3 (0.4)	1 (0.3)	
CMV IgG positive	418 (53.5)	181 (55.6)	NA
Unknown	296 (37.9)	118 (36.3)	

### T-Cell Subsets in PWH and Age Matched Controls

**FIGURE 2:**
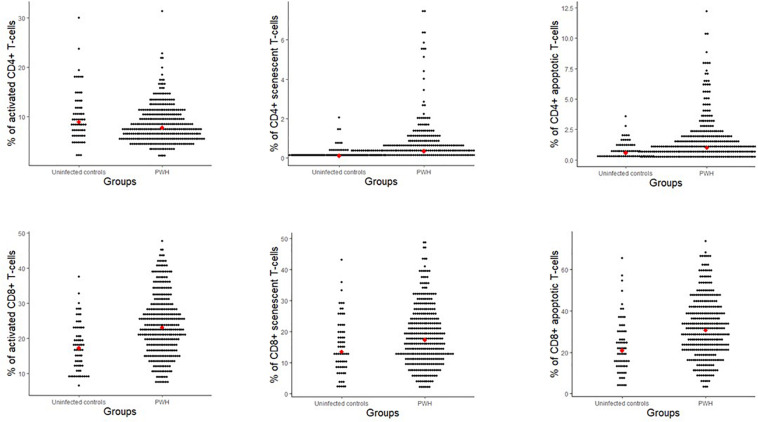
Proportions of T-cell subsets (% of CD4+ and CD8+) for people with HIV and uninfected controls matched on age. Red dot indicates the median value. Activation (CD38+ HLA-DR+), senescent (CD28-CD57+), and apoptotic (CD28-CD95+).

When comparing PWH (*n* = 325) and age-matched controls (*n* = 65), PWH had higher proportions of CD8+ activated T-cells (*p* < 0.001), CD4+ and CD8+ senescent T-cells (*p* < 0.001 and *p* = 0.008), respectively and CD4+ and CD8+ apoptotic T-cells (*p* < 0.001 and *p* < 0.001, respectively) (Figure 2). In contrast, PWH had lower proportions of CD4+ activated T-cells (*p* = 0.016) than uninfected controls (Figure 2).

**FIGURE 3:**
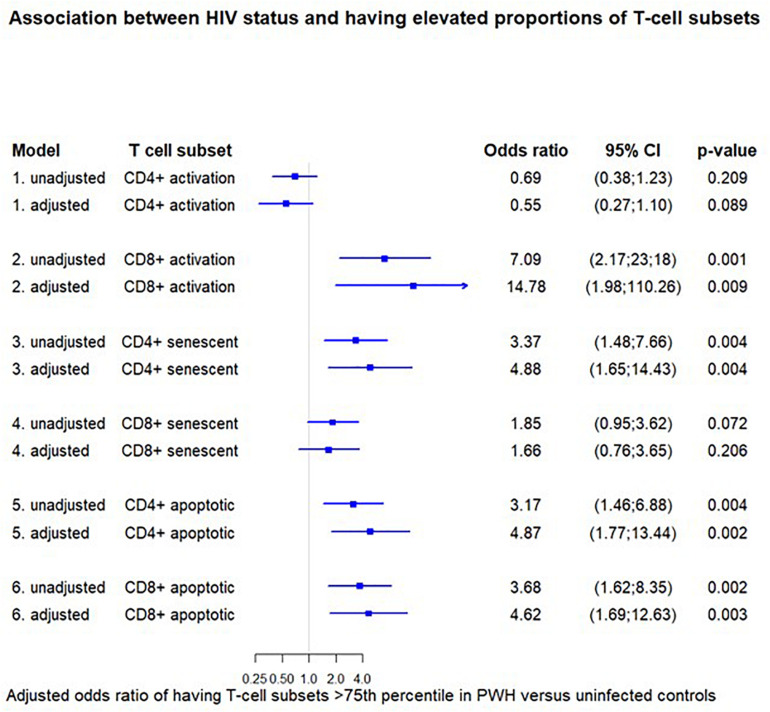
Association between HIV status and having elevated proportions of T-cell subsets. The odds ratio for having elevated proportions of the given T-cell subset (>75th percentile) in HIV-positive versus negative individuals. Each odds ratio is from a separate multivariate logistic regression analysis. The adjusted models are adjusted for age, sex, BMI group, and smoking status. Cut-off values were: CD4+ activated 10.7%, CD8+ activated 28.9%, CD4+ senescent 0.6%, CD8+ senescent 23.1%, CD4+ apoptosis 1.7%, CD8+ apoptosis 38.2. Activation (CD38+ HLA-DR+), senescent (CD28-CD57+), and apoptotic (CD28-CD95+).

When defining elevated proportion of a T-cell subsets as above the 75th percentile the cut-off values were: CD4+ activated 10.7%, CD8+ activated 28.9%, CD4+ senescent 0.6%, CD8+ senescent 23.1%, CD4+ apoptosis 1.7%, and CD8+ apoptosis 38.2%. A positive HIV status was associated with a higher adjusted odds ratio (aOR) of having elevated proportions of CD8+ activated T-cells [14.78 (1.98; 110.26), *p* = 0.009], CD4+ senescent T-cells [4.88 (1.65; 14.43), *p* = 0.004], and CD4+ and CD8+ apoptotic T-cells [4.87 (1.77; 13.44), *p* = 0.002] and [4.62 (1.69; 12.63), *p* = 0.003, respectively] after adjusting for sex, age, BMI group, and smoking status. Results from both unadjusted and adjusted analyses are shown in Figure 3.

### T-Cell Subsets and Age in PWH

In the entire group of PWH (*n* = 780), we investigated the impact of 10-year increase in age using multivariable linear regression models adjusting for sex, BMI group, and smoking status. We found that 10-year increase in the age variable was associated with 6.7 and 2.9% lower proportions of CD4+ and CD8+ activated T-cells (*p* < 0.001 and 0.028, respectively), 16.2 and 11.6% higher proportions of CD4+ and CD8+ senescent T-cells (*p* = 0.001 and *p* < 0.001, respectively), and 18.5 and 12.8% higher proportions of CD4+ and CD8+ apoptotic T-cells (*p* < 0.001 and *p* < 0.001, respectively) (Table 2). These associations were still significant after further adjusting for nadir CD4+ count and time on cART except for CD8+ activated T-cells (*p* = 0.297) (Table 2). No associations between age and T-cell subsets were found in uninfected controls (Table 2).

**TABLE 2:** Association between 10-years increase in age and proportions of T-cell subsets.

**PLWH**					**Uninfected controls**	
T-cell subsets	**Model 1**	***p***	**Model 2**	***p***	**Model 1**	***p***
CD4+ activation	−6.7% (−9.5; −3.9)	<0.001	−5.8 (−8.6; −1.9)	0.001	2.02% (−14.8; 22.2)	0.821
CD8+ activation	−2.9% (−4.8; 0.0)	0.028	−2.0 (−4.9; 1.0)	0.297	7.3% (−7.8; 24.6)	0.347
CD4+ senescent	16.2% (7.3; 27.1)	0.001	25.9 (13.9; 39.1)	<0.001	46.2% (−3.0; 120.3)	0.074
CD8+ senescent	11.6% (6.2; 16.2)	<0.001	16.2 (10.5; 22.1)	<0.001	22.14% (−4.9; 56.8)	0.122
CD4+ apoptotic	18.5% (10.5; 25.9)	<0.001	20.9 (11.6; 31.0)	<0.001	18.5% (−10.4; 58.4)	0.239
CD8+ apoptotic	12.8% (8.3; 17.4)	<0.001	16.2 (10.5; 20.9)	<0.001	24.6% (0.0; 55.3)	0.061

*Model 1 was adjusted for sex, BMI group and smoking status. Model 2 was further adjusted for CD4+ nadir and time on cART. All T-cell subsets were log transformed before analyzed. Activation (CD38+ HLA-DR+), senescent (CD28-CD57+), and apoptotic (CD28-CD95+).*

In analyses including both PWH and uninfected controls, no interaction between HIV status and age were found in models adjusting for sex, BMI group, and smoking status on any of the T-cell subsets. All *p*-values were >0.2.

### T-Cell Subsets and HIV-Specific Risk Factors

In PWH, we evaluated the association between HIV-specific factors and having elevated proportions of a T-cell subset (>75th percentile) in models adjusting for age, sex, BMI group, and smoking status. Having a CD4+/CD8+ ratio <1 was associated with a higher aOR of having elevated proportions of CD8+ activated T-cells (*p* < 0.001), CD4+ and CD8+ senescent T-cells (*p* < 0.001 and *p* < 0.001, respectively) and CD4+ and CD8+ apoptotic T-cells (*p* < 0.001 and *p* < 0.001, respectively) (Table 3). A nadir CD4+ count <200 cells/μL was associated with a lower aOR of having elevated proportions of CD8+ senescent T (*p* = 0.041) (Table 3). CMV serology was available for 484 (62%) of PWH, and 86% had a positive CMV IgG. A positive CMV IgG was not significantly associated with having elevated proportions of any of the T-cells subsets (Table 3).

**TABLE 3:** Associations between HIV-specific factors and having elevated proportions of T-cell subsets (*n* = 780).

	**CD4/CD8 ratio ≤1**				**Nadir CD4+ ≤200 cells**				CMV IgG positive*			
	**uOR (95% CI)**	***p***	**aOR (95% CI)**	***p***	**uOR (95% CI)**	***p***	**aOR (95% CI)**	***p***	**uOR (95% CI)**	***p***	**aOR (95% CI)**	***p***
CD4+ activation	0.97 (0.68; 1.37)	0.850	0.94 (0.65; 1.35)	0.727	0.85 (0.60; 1.19)	0.332	1.00 (0.69; 1.45)	0.996	1.59 (0.80; 3.16)	0.183	1.50 (0.74; 3.02)	0.257
CD8+ activation	2.38 (1.64; 3.46)	<0.001	2.35 (1.59; 3.46)	<0.001	1.10 (0.79; 1.52)	0.583	1.18 (0.83; 1.68)	0.354	0.91 (0.51; 1.61)	0.735	0.88 (0.48; 1.58)	0.660
CD4+ senescent	2.21 (1.52; 3.21)	<0.001	1.34 (1.34; 2.92)	<0.001	0.78 (0.56; 1.09)	0.148	0.66 (0.46; 0.95)	0.254	1.83 (0.95; 3.55)	0.072	1.98 (0.99; 3.95)	0.053
CD8+ senescent	2.67 (1.81; 3.94)	<0.001	2.65 (1.75; 4.00)	<0.001	0.85 (0.61; 1.19)	0.345	0.68 (0.47; 0.98)	0.041	1.20 (0.65; 2.22)	0.562	1.22 (0.63; 2.34)	0.556
CD4+ apoptotic	2.00 (1.38; 2.89)	<0.001	2.00 (1.35; 2.98)	<0.001	0.94 (0.68; 1.31)	0.731	0.76 (0.52; 1.09)	0.135	1.49 (0.78; 2.84)	0.223	1.52 (0.76; 3.02)	0.233
CD8+ apoptotic	2.64 (1.80; 3.88)	<0.001	2.61 (1.73; 3.93)	<0.001	0.96 (0.69; 1.33)	0.807	0.70 (0.48; 1.01)	0.057	1.26 (0.68; 2.33)	0.462	1.39 (0.71; 2.71)	0.331

*The adjusted odds ratio was adjusted for age, sex, BMI group and smoking status. ^∗^CMV serology was available on 484 people with HIV. Only participants with available serology was included in the analysis. uOR, unadjusted odds ratio; aOR, adjusted odds ratio; CMV, cytomegalovirus.*

## Discussion

In this study we included a large cohort of well-treated PWH with undetectable viral replication and without chronic viral hepatitis infection and used age-matched controls for comparison. We found that well-treated PWH had residual immune dysfunctions with elevated proportions of CD8+ activation, CD4+ senescence, and CD4+ and CD8+ apoptosis even after adjusting for confounders including age, sex, BMI group, and smoking status. In addition, in PWH older age was associated with an increase in T-cell senescence and apoptosis. Lastly, we found the CD4+/CD8+ ratio in PWH to be associated with elevated proportions of activated, senescent and apoptotic T-cells.

Our results support that well-treated PWH have residual immune dysfunctions since a positive HIV status was independently associated with having elevated proportions of CD8+ immune activation, CD4+ senescence, and CD4+ and CD8+ apoptosis. In contrast, no association was found between a positive HIV status and elevated proportions of CD8+ senescent T-cells. We defined elevated proportions using a cut-off above the 75th percentile to establish an upper limit of normal. With this approach small differences between PWH and uninfected controls were not investigated. Other studies have reported alterations in the T-cell subsets in treated PWH, but results have been conflicting. Thus, elevated proportions of immune activation, senescence and apoptosis have been reported (8, 10, 22, 23), while other studies found comparable proportion of activated (24, 25) or senescent T-cells in PWH and controls (8, 10, 26). However, most of the previous studies on T-cell proportions were small and did not adjust for lifestyle. Furthermore, some studies included PWH that had other risk factors for immune dysfunctions such as chronic HBV or HCV (24) or detectable HIV viral replication (9, 10). Importantly, T-cell residual immune dysfunction could contribute to the excess risk of age related comorbidity in PWH since both T-cell activation and senescence have been associated with CVD, lung disease and multimorbidity in PWH (27–30).

We found that increasing age in PWH was associated with higher proportions of senescent and apoptotic T-cells. This is consistent with other studies including PWH (31, 32) and studies conducted in the general population (17). It is likely that the elder PWH in this study had lower nadir CD4+ since European guidelines for cART treatment initiation have changed during the last 10–15 years with previous recommendations for treatment initiation at either CD4+ T cell counts of 200 or 350 cells/μL. However, since publication of the Strategic Timing of Antiretroviral Treatment (START) study in 2015, it has been recommended to treat all PWH regardless of CD4+ T-cell count (33). To reduce the potential confounding of CD4+ nadir count and treatment duration, we adjusted for these factors, and found the described associations to be robust. To our surprise, we found that increasing age was associated with lower proportions of CD4+ activated T-cells. Earlier studies have not reported an association between age and immune activation (10, 34). We speculate that the inverse association between age and activated CD4+ T-cells may be due to statin use, which may be more prevalent among older persons, and has been proposed to influence the level of immune activation (35). However, the impact of statin use among PWH is debated and we did not include statin use in our analysis (11, 36; 37). The inverse association could be due to other unknown confounders as well. We did not find an association between age and T-cell subsets in the uninfected controls which may be due to the low number of participants in this group since older age has been associated with T-cell senescence and apoptosis in the general population (38–40).

It has been suggested that HIV infection may cause accelerated immunological aging. Our data supports that HIV infection has similar effects as aging on T-cell subsets. However, our data do not support accelerated aging within PWH since we did not find an interaction between HIV status and age. This finding is in line with another study (10). In the general population an association between older age and inverted CD4+/CD8+ ratio <1 has been reported, and inverted CD4+/CD8+ ratio has been associated with altered T-cell subsets (41). In PWH, the CD4+/CD8+ ratio often remains inverted due to persistent high CD8+ T-cell count even in persons with early treatment start (42). CMV infection may contribute to CD8+ T-cell expansion and subsequently an inverted CD4+/CD8+ ratio, and high CMV seroprevalence has been described both in PWH and older uninfected persons (42–44). In our study, a CD4+/CD8+ ratio <1 was associated with having elevated proportion of CD8+ activated, CD4+ and CD8+ senescent, and CD4+ and CD8+ apoptotic T-cells in PWH. In contrast, a positive CMV IgG was not associated with elevated proportions of any of the T-cell subsets. The impact of CMV co-infection on T-cell residual immune dysfunction in PWH has been debated. Recently, CMV-specific T-cell responses but not CMV IgG level has been associated with CD8+ immune activation and senescence in PWH (13). We did not investigate the CMV specific T-cell response in this study which could have provided important information about the impact of CMV co-infection on residual immune dysfunction in PWH.

This study was limited by its cross-sectional design which does not allow for conclusions regarding causality, and results were not adjusted for multiple comparison. Furthermore, we were not able to compare the absolute count of the different T-cell populations between PWH and uninfected controls or present CD4+ T cell count or CD8+ T cell count for the uninfected controls since lymphocyte count and CD4+ and CD8+ count were not measured in the control group at inclusion. However, to the best of our knowledge, this study represents the largest study to investigating the impact of HIV status and age on T cell subsets in a well-treated cohort of PWH and uninfected controls with detailed information about both HIV-specific and traditional risk factors. Furthermore, PWH and uninfected controls were from the same geographical and socioeconomic area and all laboratory work on T-cells subsets were done uniformly in the same laboratory and analyzed by the same operator.

In summary, this study showed that residual T-cell immune dysfunction is found in well-treated PWH with undetectable viral replication and absence of chronic HBV and HCV co-infection. T-cell senescence and apoptosis increased with age, and CD4/CD8+ ratio was a strong predictor of T-cell activation, senescence and apoptosis in PWH. However, no evidence of accelerated immunological aging was found. Residual immune dysfunction may contribute to the excess risk of age-related comorbidity in PWH, and further studies investigating the impact of residual T-cell dysfunction on comorbidity in PWH are warranted.

## Data Availability Statement

The dataset analyzed during the current study is available from the corresponding author on reasonable request.

## Ethics Statement

The studies involving human participants were reviewed and approved by Committee on Health Research Ethics of the Capital Region of Denmark (H-8-2014-004). The patients/participants provided their written informed consent to participate in this study.

## Author Contributions

MH-S, HU, JG, and SN designed the study. MH-S, RT, TB, SA, BN, and SN were responsible for data collection. MH-S, YZ, JT, HH, and HU performed the laboratory analyses. MH-S, AM, and SN were responsible for the statistical analyses. All authors interpreted the data. MH-S drafted the manuscript. All authors have critically revised and approved the final version.

## Conflict of Interest

RT reports grant from Rigshospitalet Research Council and traveling grant from Gilead. AM reports consultancy fees, lecture fees, travel support and/or honorarium from ViiV, Gilead and Eiland Bonnin PC. SN reports unrestricted research grants from Novo Nordisk Foundation, Lundbeck Foundation, and Rigshospitalet Research Council. Traveling grants from Gilead. Advisory board activity for Gilead and GSK/ViiV. MH-S, YZ, JT, HH, TB, SA, BN, HU, and JG declares no conflict of interest.
